# Glycerol-3-Phosphate Metabolism in Wheat Contributes to Systemic Acquired Resistance against *Puccinia striiformis* f. sp. *tritici*

**DOI:** 10.1371/journal.pone.0081756

**Published:** 2013-11-29

**Authors:** Yuheng Yang, Jing Zhao, Peng Liu, Huijun Xing, Chaochao Li, Guorong Wei, Zhensheng Kang

**Affiliations:** State Key Laboratory of Crop Stress Biology for Arid Areas and College of Plant Protection, Northwest A&F University, Yangling, Shaanxi, People’s Republic of China; Nanjing Agricultural University, China

## Abstract

Glycerol-3-phosphate (G3P) is a proposed regulator of plant defense signaling in basal resistance and systemic acquired resistance (SAR). The *GLY1*-encoded glycerol-3-phosphate dehydrogenase (G3PDH) and *GLI1*-encoded glycerol kinase (GK) are two key enzymes involved in the G3P biosynthesis in plants. However, their physiological importance in wheat defense against pathogens remains unclear. In this study, quantification analysis revealed that G3P levels were significantly induced in wheat leaves challenged by the avirulent *Puccinia striiformis* f. sp. *tritici* (*Pst*) race CYR23. The transcriptional levels of *TaGLY1* and *TaGLI1* were likewise significantly induced by avirulent *Pst* infection. Furthermore, knocking down *TaGLY1* and *TaGLI1* individually or simultaneously with barley stripe mosaic virus-induced gene silencing (BSMV-VIGS) inhibited G3P accumulation and compromised the resistance in the wheat cultivar Suwon 11, whereas the accumulation of salicylic acid (SA) and the expression of the SA-induced marker gene *TaPR1* in plant leaves were altered significantly after gene silencing. These results suggested that G3P contributes to wheat systemic acquired resistance (SAR) against stripe rust, and provided evidence that the G3P function as a signaling molecule is conserved in dicots and monocots. Meanwhile, the simultaneous co-silencing of multiple genes by the VIGS system proved to be a powerful tool for multi-gene functional analysis in plants.

## Introduction

Plants are continuously exposed to a vast number of potential pathogenic microbes. However, most plants are immune against a mass of microbial invaders [1,2]. Plants rely on the innate immunity of each cell and the signaling pathways activated at infection sites, which both constitute the plant immune system [3]. There are two strategies that plants utilize to detect pathogenic microbes. On the cell surface of the host plants, conserved pathogen-associated molecular patterns (PAMPs) are recognized by pattern recognition receptors (PRRs), such as fungal chitin or bacterial flagellin. Stimulation of PRRs leads to PAMP-triggered immunity (PTI) [4]. Plants have evolved various nucleotide-binding (NB) and leucine-rich repeat (LRR) proteins, which are the most common disease resistance (*R*) proteins in response to pathogen effectors by intracellular receptors. NB-LRR proteins recognize effectors and initiate effector-triggered immunity (ETI) [5].

The function of both PTI and ETI in plant resistance against pathogens requires intricate signaling networks, which involve various phytohormones that regulate signal transduction pathways [6,7]. Previous evidence suggests that the components of primary metabolism, fatty acids, and their derivatives are important signaling molecules in plant defense; these compound interact with endogenous phytohormones, including salicylic acid (SA), jasmonic acid (JA) and ethylene (ET) [6,8]. For instance, the proper localization of NPR1, a key component of the SA signaling pathway in *Arabidopsis*, is dependent on the activity of glucose-6-phosphate dehydrogenase [9]. The reduction of oleic acid (18:1) can induce expression of the turnip crinkle virus (TCV) resistance gene *HRT*, which responds to SA and confers resistance to TCV in *Arabidopsis* [10].

Glycerol-3-phosphate (G3P) is an obligatory component and precursor of all plant glycerolipid biosynthesis; it also participates in fatty acid biosynthesis. Previous studies reported that G3P is essential for basal resistance and systemic acquired resistance (SAR) in *Arabidopsis* [8,11–14]. Plant G3P is derived via the glycerol kinase (GK)-mediated phosphorylation of glycerol or the NAD-dependent G3P dehydrogenase (G3Pdh)-mediated reduction of dihydroxy-acetone phosphate (DHAP) [11]. In *Arabidopsis*, the *GLY1*/*SFD1* (encodes G3Pdh) and *GLI1*/*NHO1* (encodes GK) genes contribute to resistance against hemibiotrophic fungi (*Colletotrichum higginsianum*) and bacteria (*Pseudomonas syringae* DC 3000) [8,11,15]. Furthermore, *GLI1* is nonspecifically required for nonhost resistance (NHR) to bacterial and fungal pathogens [16,17].

Although *GLY1* and *GLI1* are required for resistance to fungal and bacterial pathogens in *Arabidopsis*, their roles in monocots have not been demonstrated. In this study, we isolated and characterized a set of G3Pdh homologous genes and a GK homologous gene from wheat plants challenged with *Puccinia striiformis* Westend f. sp. *tritici* Erikss. (*Pst*). *Pst* is an obligate biotrophic basidiomycete that is one of the top ten plant-pathogenic fungi [18]. Quantification analysis revealed that G3P levels, as well as the expression of *TaGLY1* and *TaGLI1*, could be induced in wheat leaves challenged by the avirulent *Pst* race CYR23. Furthermore, the barley stripe mosaic virus-induced gene silencing (BSMV-VIGS) system was selected to evaluate the functions of G3P in *TaGLY1* and *TaGLI1* resistance.

## Results

### G3P levels changed are associated with wheat against Pst

**Figure 1:**
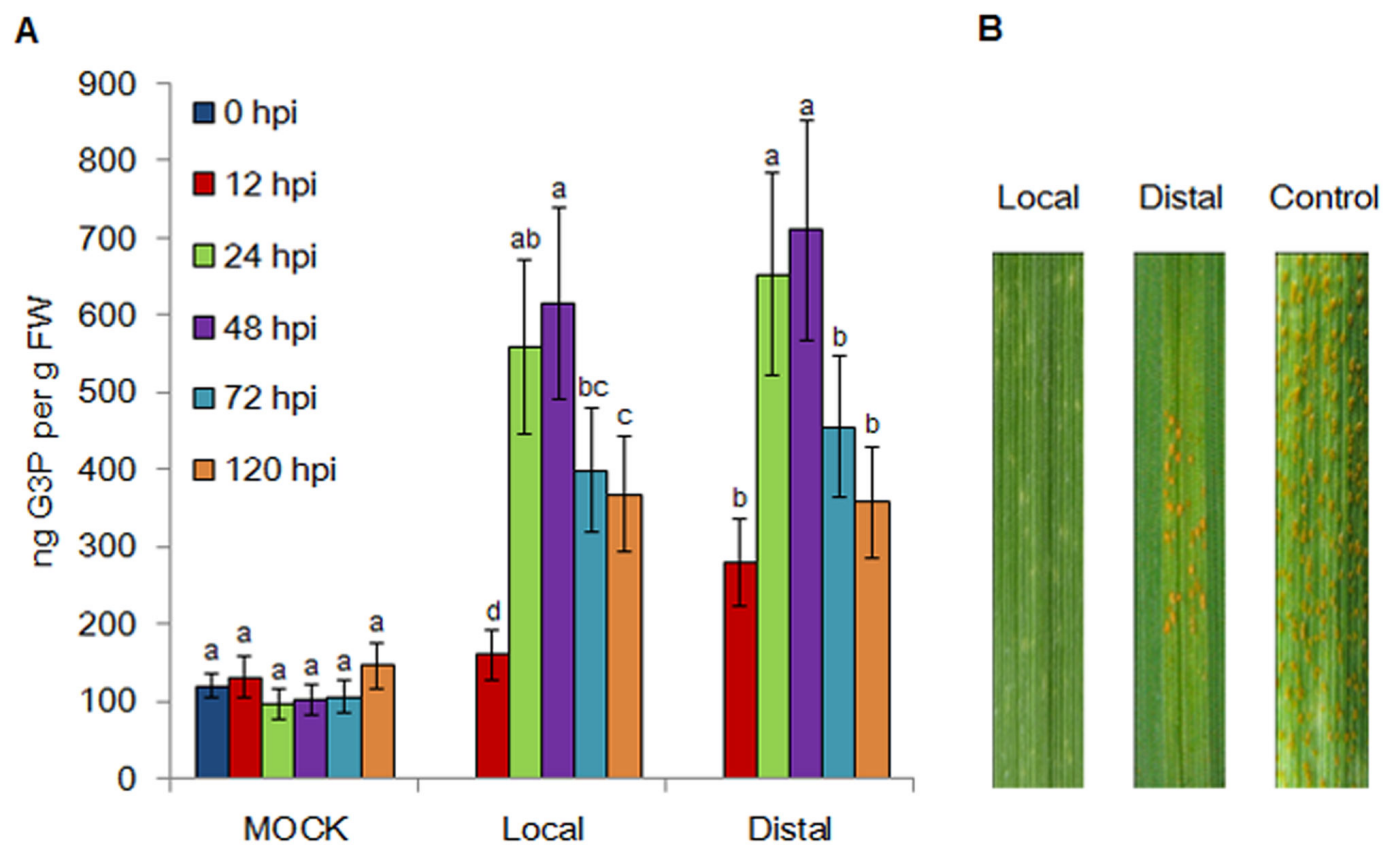
Changing G3P levels are associated with wheat against *Puccinia striiformis* f. sp. *tritici*. (A) G3P levels were increased by *Pst* infection in wheat primary (local) leaves were inoculated with the avirulent race CYR23, whereas the distal leaves were inoculated with the virulent CYR31 48 h later. Leaf tissues were sampled for both local and distal leaves at 0, 12, 24, 48, 72 and 120 hpi. Mock was wheat leaves treated with water. FW indicates fresh weight. ANOVA was performed to determine the differences between each treatment. Superscripts with the same letter indicate that values are not significantly different at *P* < 0.01. (B) Disease symptoms in Pst-inoculated wheat leaves at 15 days after inoculation. The primary (local) leaves were inoculated with CYR23, whereas the distal leaves were inoculated with CYR31 48 h later. Control was inoculation of primary leaves with water and then the distal leaves with CYR31 48 h later.

To determine whether G3P is involved in the SAR of wheat against *Pst*, we inoculated the primary leaves with the *Pst* race CYR23 (incompatible interaction) and the distal leaves with CYR31 (compatible interaction) after 48 h. Subsequently, the G3P content in the primary and distal leaves of the wheat cultivar Suwon 11 (Su11) seedlings were compared. The G3P content was strongly increased in both local and distal leaves after 12 hours post-inoculation (hpi), as compared with the water inoculated control. Two respective peaks (more than sixfold) at 48 hpi were observed for the primary and distal leaves (Figure 1A). After 15 days post-inoculation (dpi) of CYR31, reduced rust sporulation was observed in distal leaves, as compared with the controls that were inoculated on primary leaves with water and then the distal leaves with CYR31 48 h later. (Figure 1B). The results indicated that G3P is a potential signaling molecule involved in the SAR of wheat against *Pst*.

### Cloning and transcriptional responses of wheat G3Pdh isoforms and GK against Pst infection

Four genes encode different *G3Pdh* isoforms and only one gene encodes GK in the *Arabidopsis* and rice genomes (Table 1). Therefore, TBLASTN was applied with stringent criteria to identify probable orthologous wheat sequences in GenBank (http://www.ncbi.nlm.nih.gov/genbank). Four *G3Pdh* genes and a GK gene were assembled with a set of sequences that were identified in the wheat EST database. Subsequently, all assembled cDNA fragments were amplified by reverse transcription using the primers in Table S1.

**Table 1:** Genes for G3P biosynthesis in wheat that correspond with the respective genes in *Arabidopsis* and rice.

Wheat	Arabidopsis	Rice
*TaGPDH1* (GenBank accession No. KC953025)	At2g41540 (71%), At3g07690 (69%)	Os01g58740 (76%), Os01g71280 (89%), Os05g41590 (76%)
*TaGPDH2* (GenBank accession No. KC953026)	At3g10370 (72%)	ND
*TaGPDH3* (GenBank accession No. KC953027)	At5g40610 (71%)	Os01g74000 (88%)
*TaGLY1* (GenBank accession No. KC527592)	At2g40690 (68%)	Os07g12640 (89%)
*TaGLI1* (GenBank accession No. KC244204)	At1g80460 (72%)	Os04g55410 (87%)

Percentages of gene identities were showed in parentheses, respectively.

ND = no ortholog was detected.

**Figure 2:**
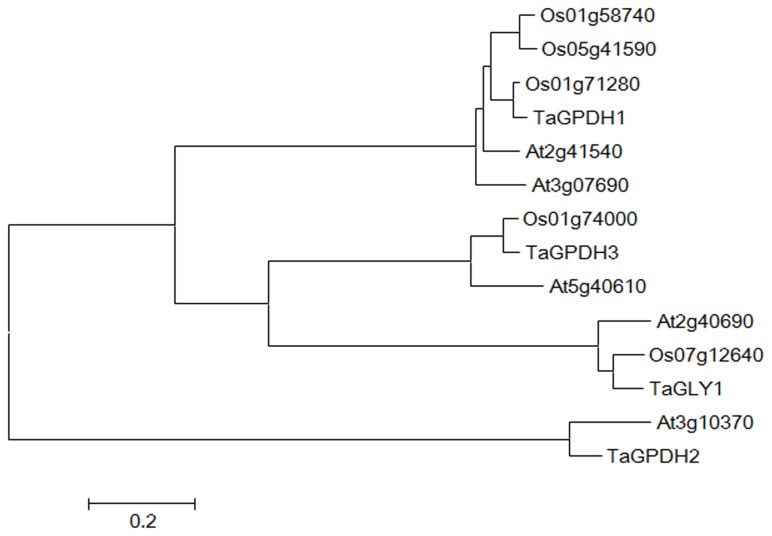
Phylogenetic analyses between four TaG3Pdhs and G3Pdh member proteins from *Arabidopsis* and rice. Amino acid sequences were aligned using ClustalW and constructed phylogenetic tree using neighbor-joining method by molecular evolutionary genetics analysis (MEGA). The four TaG3Pdhs were grouped into four units.

After analyzing the open reading frames (ORFs) of all obtained cDNA sequences, the TaG3Pdhs and selected G3Pdh proteins were used to construct a neighbor-joining phylogenetic tree (Figure 2). According to Figure S1, one of the wheat *G3Pdh* encoding genes was named *TaGLY1* (GenBank accession No. KC527592) based on its amino acid sequence homology with AtGLY1 (At2g40690), whereas the other three wheat *G3Pdh* genes were named *TaGPDH1-3* (Table 1). Meanwhile, the wheat GK encoding gene was identified as *TaGLI1* (GenBank accession No. KC244204) based on its amino acid sequence homology with AtGLI1 (At1g80460) (Figure S2).

**Figure 3:**
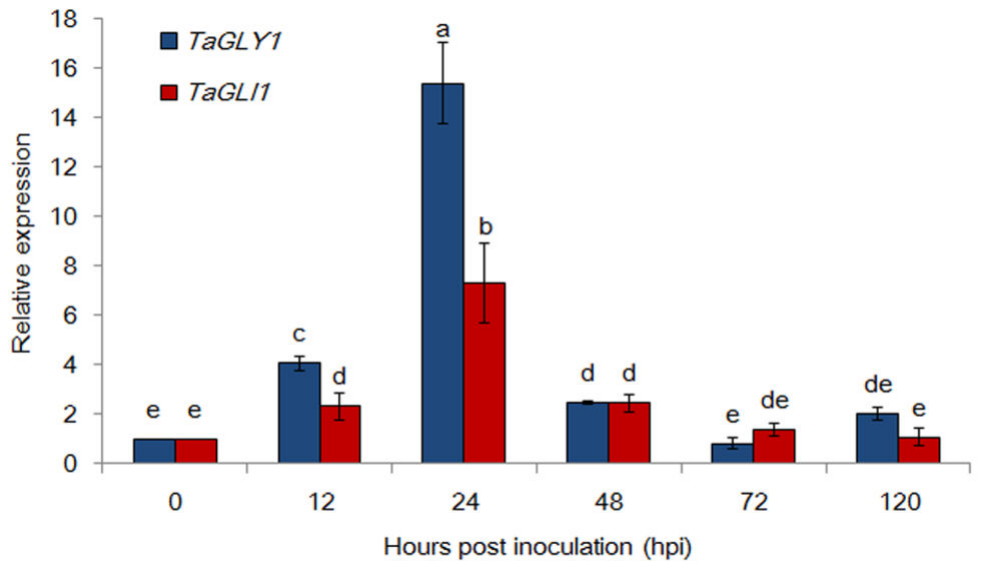
Relative transcriptional changes of *TaGLY1* and *TaGLI1* induced by *Pst* infection in wheat leaves after inoculation with the avirulent pathogen CYR23. Leaf tissues were sampled for both inoculated and mock-inoculated plants at 0, 12, 24, 48, 72 and 120 hpi. The relative expression levels of *TaGLY1* and *TaGLI1* were calculated using the comparative threshold (2^–ΔΔC^_*T*_) method. ANOVA was performed to determine the differences between each treatment. Superscripts with the same letter indicate that values are not significantly different at *P* < 0.01. The mean value and standard deviation of gene expression were calculated from three independent biological replications.

When wheat cultivar Su11 seedlings were challenged with the avirulent *Pst* race CYR23, four *TaG3Pdhs* and *TaGLI1* had different expression levels. The expression of *TaGLY1* and *TaGLI1* was clearly upregulated at 12 hpi, as compared with the control, with more than a 15-fold and 8-fold (Figure 3) increase, respectively, than the controls at 24 hpi. The expression levels eventually decreased to normal at 48 hpi. Both *TaGPDH2* and *TaGPDH3* were similarly induced at 24 hpi, but at more moderate levels (fourfold and fivefold, respectively; Figure S3). However, the expression levels of *TaGPDH1*was not significantly induced by *Pst* infection (Figure S3). These results suggested that *TaGLY1* and *TaGLI1* may be involved in G3P synthesis during *Pst* infection, although other *G3Pdh* genes also contribute to G3P accumulation

### Response to Pst infection after knocking down TaGLY1 and TaGLI1 in wheat

**Figure 4:**
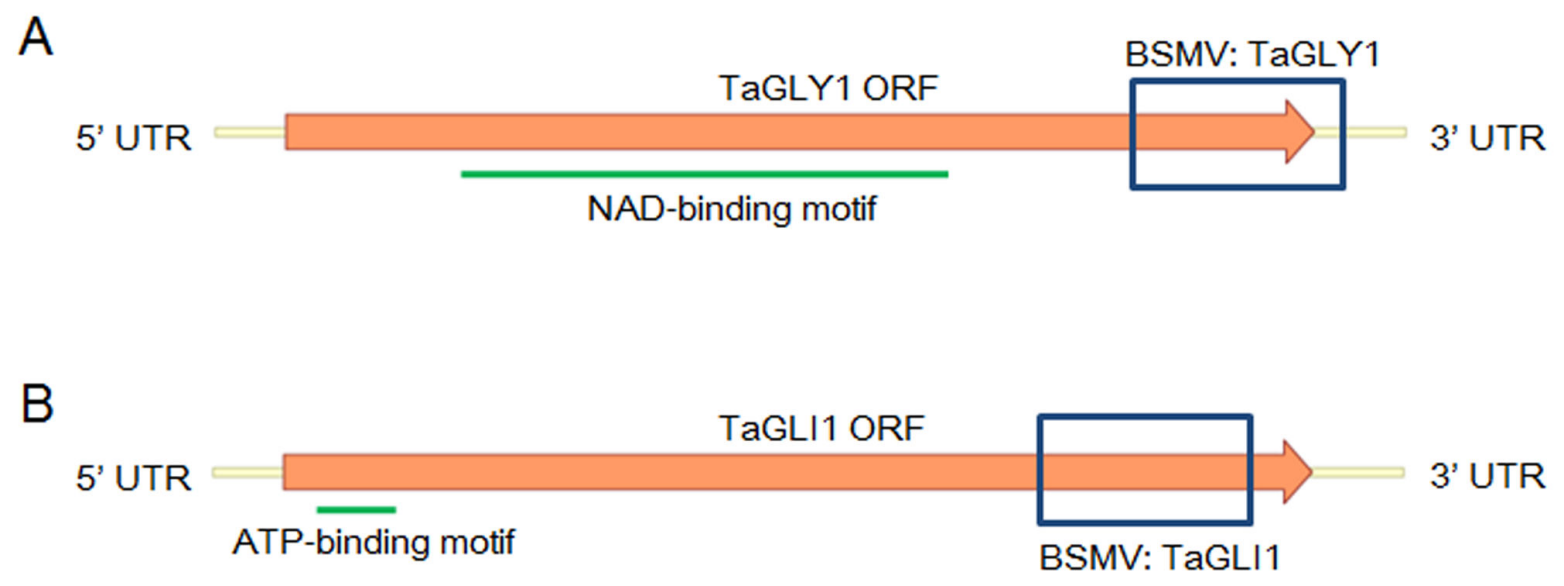
Primers designed for knocking down *TaGLY1* and *TaGLI1* expression. Two virus-induced gene silencing (VIGS) sites were designed on the *TaGLY1* (A) and *TaGLI1* (B) genes to prepare the specific knock-down fragments. The knock-down fragments are boxed.

**Figure 5:**
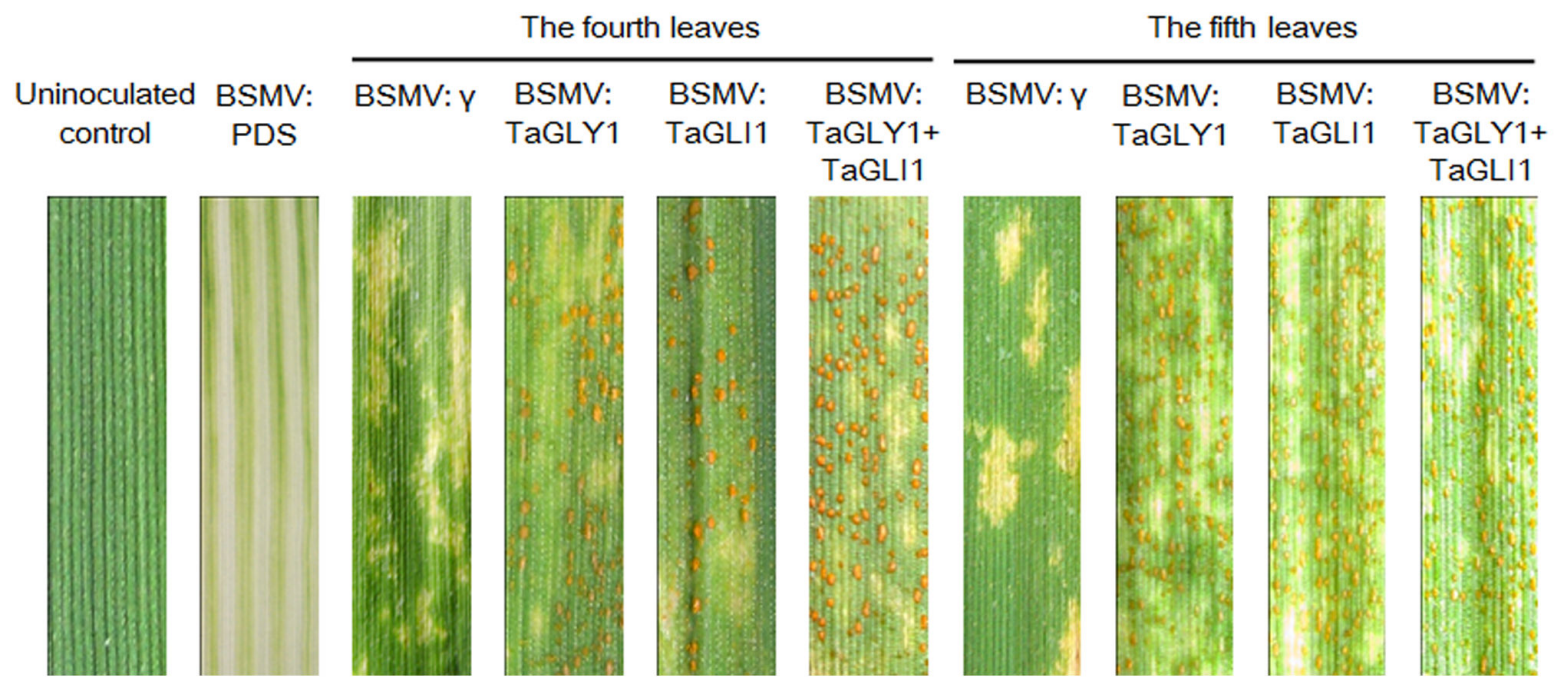
Functional characterization of *TaGLY1* and *TaGLI1* in response to stripe rust infection using the BSMV-VIGS system. Disease symptoms were observed at 14 dpi on the fourth and fifth leaves of wheat plants inoculated with the BSMV virus and the avirulent pathogen CYR23. Photo-bleaching symptoms were observed at 14 dpi on wheat seedlings when *TaPDS* was silenced with BSMV:PDS. Mock trials of wheat leaves were BSMV:γ control plants infected with CYR23.

Based on the changes in *TaGLY1* and *TaGLI1* expression during *Pst* inoculation, the BSMV-VIGS system was employed to knock down the transcription of *TaGLY1* and *TaGLI1* and to further investigate their functions in response to *Pst* infection (Figure 4). The feasibility and silencing efficiency of the BSMV-VIGS system in Su11 was tested using the wheat phytoene desaturase (TaPDS) as a positive control. At 12 dpi with BSMV:TaPDS, photo-bleaching symptoms were observed on wheat seedlings when *TaPDS* was silenced (Figure 5). Therefore, the BSMV-VIGS system could be used for assessing the potential roles of *TaGLY1* and *TaGLI1* in wheat resistance against *Pst* infection. Under the same conditions, at 12 dpi with the BSMV:TaGLY1 and BSMV:TaGLI1 recombinant vectors, the mild mosaic and chlorotic phenotypes were apparent in fourth and fifth leaves of Su11 seedlings. Similar results were observed with the control seedlings inoculated with BSMV:γ. To study the relationship between *TaGLY1* and *TaGLI1* in *Pst* resistance, two recombinant vectors were mixed together and rub-inoculated onto Su11 seedlings.

To further study whether *TaGLY1*-, *TaGLI1*- and *TaGLY1/TaGLI1-*knockdown plants compromise SAR against *Pst*, in the subsequent experiments, the fourth (local) leaves were inoculated with CYR23, and then the fifth (distal) leaves were challenged with CYR23 48 h later. As shown in Figure 5, a conspicuous hypersensitive response (HR) was elicited by *Pst* inoculation in control leaves and leaves with gene silencing. Various numbers of *Pst* uredia were produced around the necrotic spots on the fourth and fifth leaves infected with BSMV:TaGLY1 and BSMV:TaGLI1, as compared with the control plants (Figure 5). More uredia were noticeably observed on the BSMV:TaGLY1/TaGLI1 plant leaves than those with single gene silencing at 14 dpi with the avirulent *Pst* race (Figure 5). Quantification of the percentages of leaf segment areas covered with *Pst* uredia gave similar results, wherein the uredia covered 6.65%, 4.50% and 12.60% of the surface area in leaves with knocked-down segments of *TaGLY1*-, *TaGLI1*- and *TaGLY1*/*TaGLI1*, respectively (Table 2; Figure S4). These observations suggested that *TaGLY1* and *TaGLI1* may have synergistic effects on their respective functions. By contrast, no significant differences (*P* < 0.01) were observed in the percentages of the fifth leaf segment areas covered with *Pst* uredia between the leaves with knocked-down *TaGLY1*-, *TaGLI1*- and *TaGLY1*/*TaGLI1* (Table 2; Figure 5; Figure S4), Therefore, the knockdown of *TaGLY1*, *TaGLI1* or *TaGLY1*/*TaGLI1* significantly weakened wheat SAR against *Pst* infection.

**Table 2:** Quantification of the percentages of leaf areas covered with *Pst* uredia at 14 dpi, when the transcription of *TaGLY1* and *TaGLI1* was repressed.

Treatment	Percentages of leaf areas covered with *Pst* (%)
BSMV: γ	ND
*Local leaves*	
BSMV: TaGLY1	6.65 ± 0.79 **^*b*^**
BSMV: TaGLI1	4.50 ± 0.23 **^*c*^**
BSMV: TaGLY1/TaGLI1	12.60 ± 0.15 **^*a*^**
*Distal leaves*	
BSMV: TaGLY1	6.67 ± 0.24 **^*b*^**
BSMV: TaGLI1	6.16 ± 0.35 **^*b*^**
BSMV: TaGLY1/TaGLI1	7.04 ±0.52 **^*b*^**

Wheat local and distal leaves were infected with BSMV:γ, BSMV:TaGLY1, BSMV:TaGLI1, or BSMV:TaGLY1/TaGLI1, followed by CYR23 inoculation.

Percentage of leaf areas covered with *Pst* was categorized using color range tool of Adobe Photoshop software.

ANOVA was performed to determine the differences between each treatment. Superscripts with the same letter indicate that values are not significantly different at *P* < 0.01.

ND = not detected.

**Figure 6:**
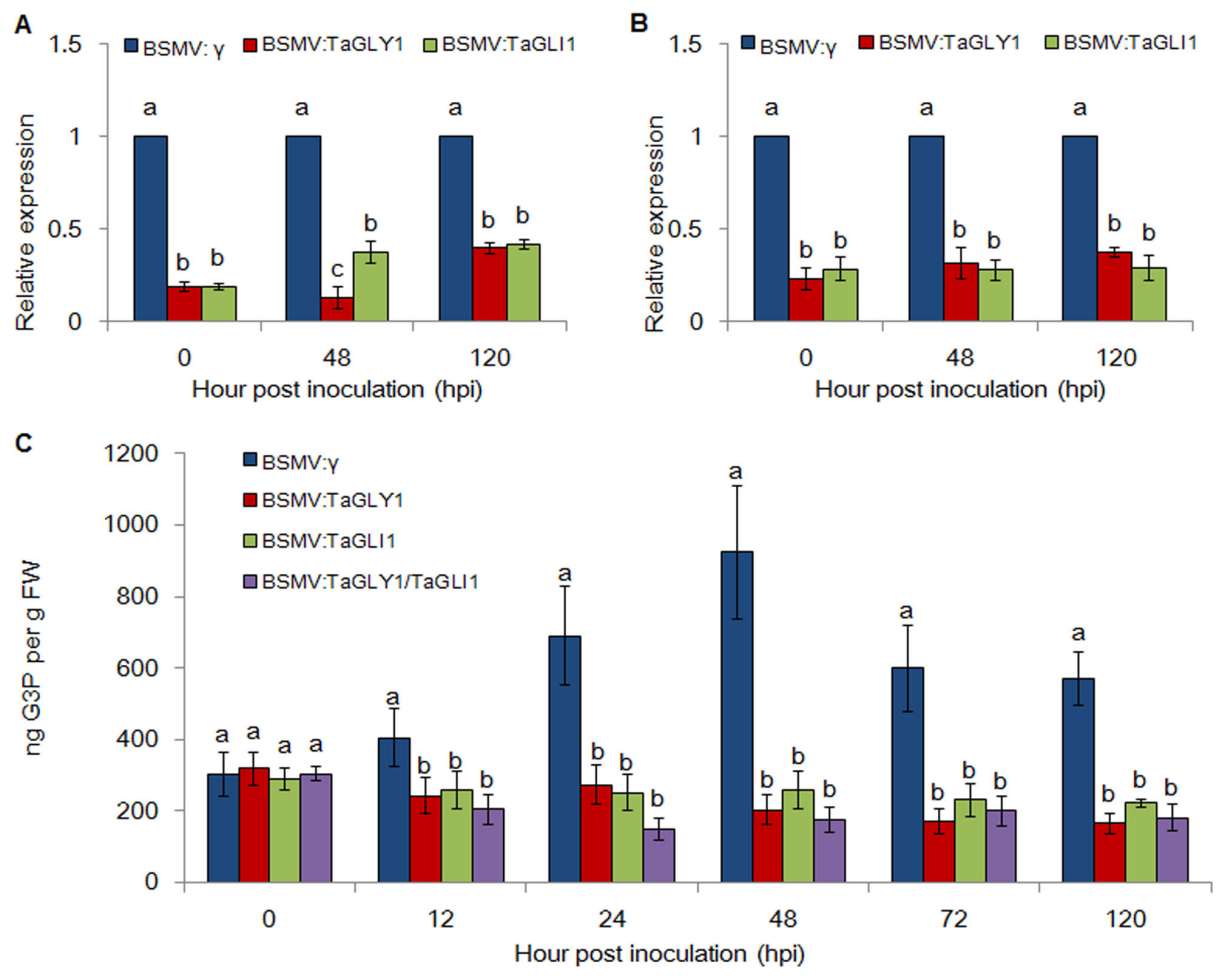
Relative transcript levels of *TaGLY1* and *TaGLI1* assayed in knocked-down wheat leaves individually in TaGLY1-silenced plants or TaGLI1-silenced plants (A) or simultaneously in TaGLY1/TaGLI1-silenced plants (B) at 0, 48 and 120 hpi. (C) The G3P levels in distal leaves of VIGS plants at 0, 12, 24, 48, 72 and 120 hpi after they were inoculated with the avirulent race CYR23. ANOVA was performed to determine the differences between each treatment. Superscripts with the same letter indicate that values are not significantly different at *P* < 0.01. The mean value and standard deviation of gene expression were calculated from three independent biological replications.

To determine the silencing efficiency of *TaGLY1* and *TaGLI1* in plants that had been infected with recombinant vectors, the relative expression levels of *TaGLY1* and *TaGLI1* in the infected leaves were detected by qRT-PCR. Compared with the BSMV:γ leaves, the expression of the *TaGLY1* transcript in the infected BSMV:TaGLY1 leaves was reduced by 81.6%, 87.1% and 60.3% after infection with avirulent *Pst* at 0, 48 and 120 hpi, respectively, whereas the corresponding *TaGLI1* transcript expression in infected BSMV:TaGLI1 leaves was reduced by 81.3%, 62.5% and 58.3%, respectively (Figure 6A). Approximately 76.9%, 68.6% and 62.8% of the *TaGLY1* transcripts and 67.5%, 72.3% and 71.0% of the *TaGLI1* transcripts were suppressed for each respective time point in the *TaGLY1*/*TaGLI1* leaves (Figure 6B). Meanwhile, the expression of *TaGPDH1-3* was not suppressed in VIGS plants (Figure S5). The silencing efficiency further confirmed that the enhanced susceptible phenotypes observed on the leaves inoculated with the avirulent *Pst* race were due to the silencing of *TaGLY1* and *TaGLI1.*

To confirm whether the knock-down of *TaGLY1*, *TaGLI1* and *TaGLY1*/*TaGLI1* could interrupt G3P accumulation, we quantified the G3P content in VIGS plants. In the control BSMV:γ leaves inoculated with the avirulent *Pst* race, the G3P level increased at 12 hpi and peaked at 48 hpi (Figure 6C). By contrast, the G3P levels declined significantly (*P* < 0.01) in leaves with knocked-down *TaGLY1*, *TaGLI1* and *TaGLY1/TaGLI1* (Figure 6C). Thus, G3P accumulation stopped because *TaGLY1* and *TaGLI1* were silenced.

### Histological changes of Pst growth and host response

**Figure 7:**
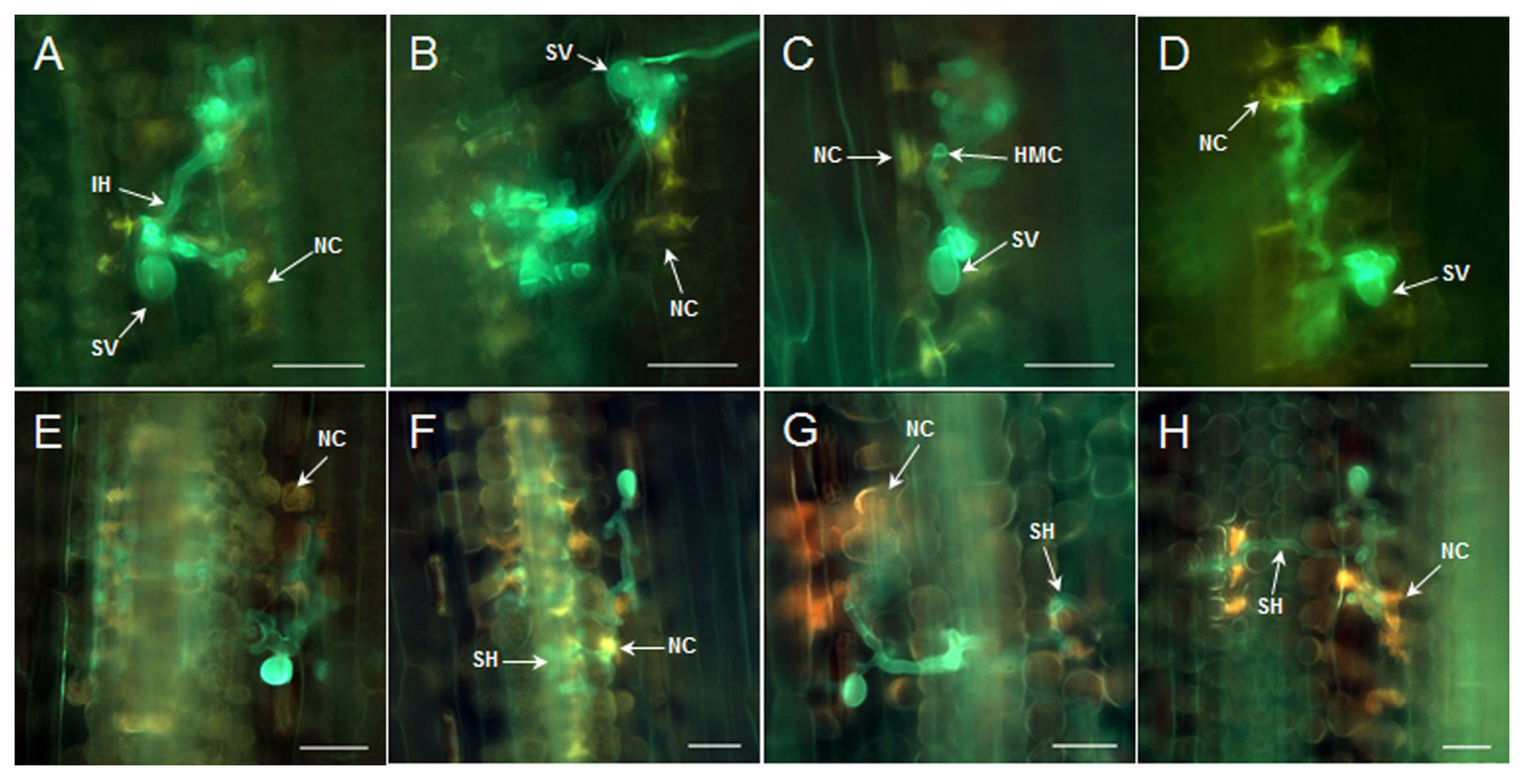
Histological observation of fungal and host cell death in BSMV-infected wheat leaves after inoculation with the avirulent race CYR23. Leaf tissues were sampled at 48 and 120 hpi. A to D, Leaves infected with BSMV:γ (A), BSMV:TaGLY1 (B), BSMV:TaGLI1 (C) and BSMV:TaGLY1/TaGLI1 (D) and inoculated with CYR23 at 48 hpi. E to H, Leaves infected with BSMV:γ (E), BSMV:TaGLY1 (F), BSMV:TaGLI1 (G) and BSMV:TaGLY1/TaGLI1 (H) and inoculated with CYR23 at 120 hpi. SV, substomatal vesicle; HMC, haustorial mother cell; IH, infected hypha; SH, secondary hypha; NC, necrotic cell. Bars, 50 μm.

To determine the histological changes associated with enhanced susceptibility to *Pst* when *TaGLY1* and *TaGLI1* were knocked-down, leaf segments from at least three plants inoculated with CYR23 were obtained for microscopic examination. Two time points (48 and 120 hpi) were chosen for comparison because the necrotic host cells start increasing and the secondary hyphae start developing at 48 hpi in the incompatible interaction [19]. As shown in Table 3 and Figure 7, significant differences (*P* < 0.05) in the necrotic areas were observed in the BSMV:TaGLY1, BSMV:TaGLI1 or BSMV:TaGLY1/TaGLI1 plants, as compared with the BSMV:γ plants at infection sites within 48 hpi to 120 hpi. Moreover, the infection site areas were obviously larger (*P* < 0.01) in plants with knocked-down *TaGLY1*-, *TaGLI1*- and *TaGLY1*/*TaGLI1* than in the control treatments at 72 and 120 hpi (Table 3; Figure 7). Therefore, the knock-down of *TaGLY1* and *TaGLI1* could reduce wheat resistance to *Pst*, thereby causing the larger hyphal growth that was partially related to HR in plant cells.

**Table 3:** Histological observations of leaves with knocked-down *TaGLY1* and *TaGLI1* during the incompatible interactions between wheat and *Pst*.

Treatment	Necrotic area per infection site (μm^2^)		Infection site areas (μm^2^)	
	48 hpi	120 hpi	48 hpi	120 hpi
BSMV: γ	1531.53 ^a^	2889.66 ^a^	1038.96 ^a^	2335.98 ^B^
BSMV: TaGLY1	1461.89 ^a^	2067.68 ^b^	1339.09 ^a^	4483.32 ^A^
BSMV: TaGLI1	1841.85 ^a^	2656.33 ^ab^	1261.52 ^a^	4024.20 ^AB^
BSMV: TaGLY1/TaGLI1	1813.76 ^a^	2274.74 ^b^	1310.39 ^a^	4976.46 ^A^

Wheat leaves were infected with BSMV:γ, BSMV:TaGLY1, BSMV:TaGLI1, or BSMV:TaGLY1/TaGLI1, followed by CYR23 inoculation.

The length and width of each necrotic spot and infection site were calculated using the DP-BSW software.

ANOVA was performed to determine the differences between each treatment. Capital letters as superscripts indicate that *P* < 0.01, whereas lowercase letters mean *P* < 0.05.

### Compromised resistance to Pst is associated with SA defection

**Figure 8:**
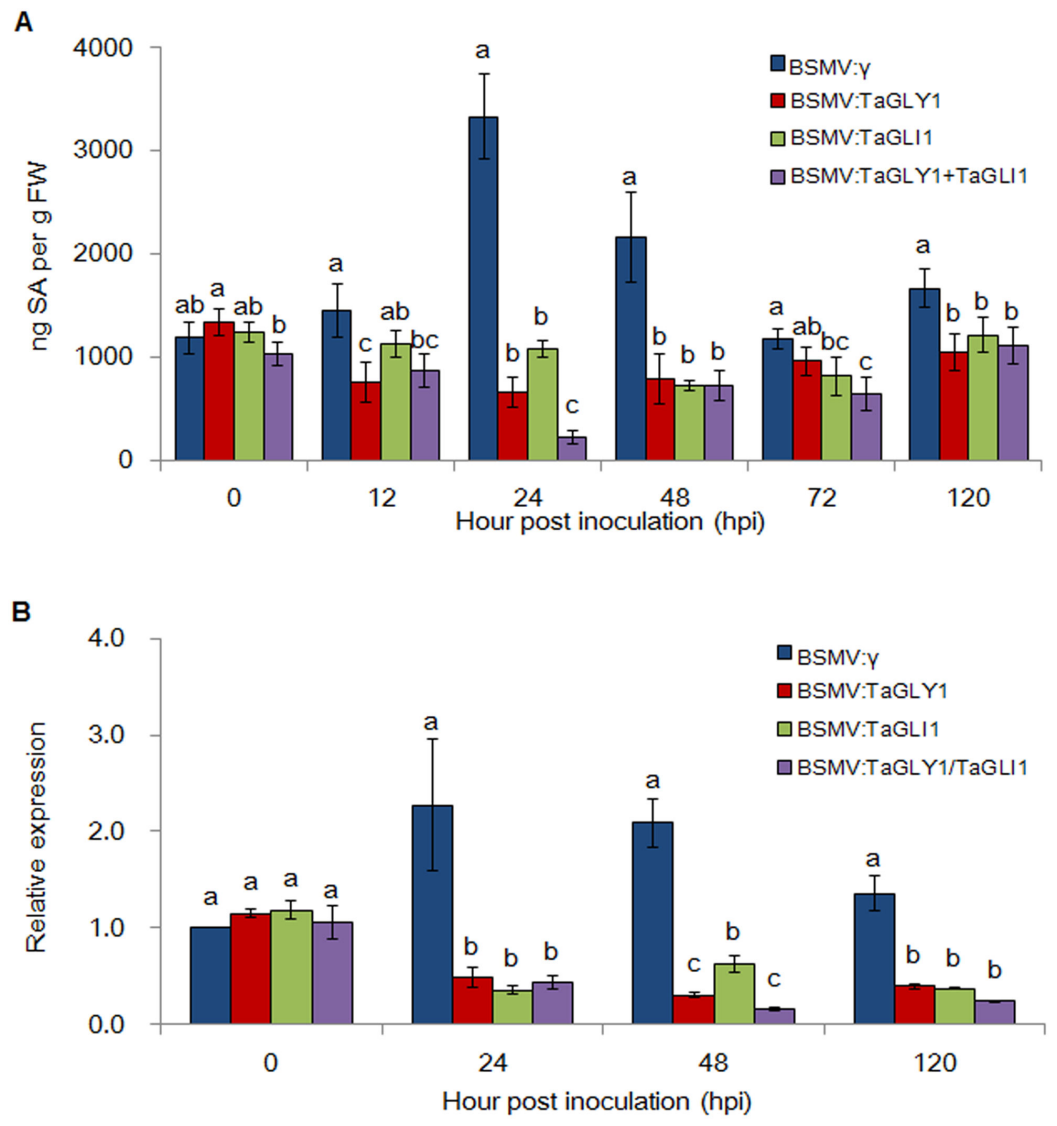
*TaGLY1* and *TaGLI1* are involved in the SA signaling pathway during wheat–*Pst* interaction. (A) SA levels in distal leaves of VIGS plants at 0, 12, 24, 48, 72 and 120 hpi after they were inoculated with the avirulent race CYR23. (B) Relative expression of the SA marker gene *TaPR1* in leaves with knocked-down *TaGLY1*, *TaGLI1*, and *TaGLY1*/*TaGLI1* at 0, 48 and 120 hpi after they were inoculated with CYR23. FW indicates fresh weight. ANOVA was performed to determine the differences between each treatment. Superscripts with the same letter indicate that values are not significantly different at *P* < 0.01.

Given that *GLY1* and *GLI1* mediated G3P synthesis is essential in SAR [11], the involvement of *TaGLY1* and *TaGLI1* in *Pst* resistance was speculated to be associated with the SA signaling pathway. To determine if knocking-down *TaGLY1*, *TaGLI1* and *TaGLY1*/*TaGLI1* can affect SA balance in wheat, thereby leading to enhanced resistance to the stripe rust fungus, we further compared the SA accumulation in *Pst* inoculated tissues of VIGS plants to the controls. The SA level was slightly increased at 12 hpi and peaked with a twofold increase at 24 hpi (Figure 8A). However, in leaves with knocked-down *TaGLY1* and *TaGLI1*, SA was obviously decreased at 12 hpi, which further declined to its lowest level at 24 and 48 hpi, respectively. After 72 hpi, the SA content returned to normal levels (Figure 8A). In BSMV:TaGLY1/TaGLI1 plants, SA accumulation showed similar tendencies with the single-silencing plants and displayed a superposition effect (Figure 8A). In addition, the SA-induced marker gene *TaPR1* (GenBank accession No. AAK60565) was selected for real-time PCR analysis. As shown in Figure 8B, the transcriptional accumulation of *TaPR1* was significantly reduced within 48 and 120 hpi in leaves with knocked-down *TaGLY1*, *TaGLI1* and *TaGLY1*/*TaGLI1*, as compared with the BSMV:γ controls. These results further indicated that *TaGLY1* and *TaGLI1* were involved in the SA pathway in response to the avirulent *Pst* race, with an apparent synergistic effect.

## Discussion

G3P has been reported to confer basal resistance to *Colletotrichum higginsianum* [8,15] and SAR by facilitating the systemic translocation of DIR1 in Arabidopsis [11]. In this study, we measured the G3P accumulation in wheat infected by *Pst*. Quantification assays revealed that G3P levels were significantly increased in both avirulent pathogen inoculated leaves and distal leaves. Meanwhile, the increased G3P accumulation was accompanied by induced SAR, thereby indicating that G3P-mediated SAR was present in gramineous plants.

Furthermore, the cloned *TaGLY1* and *TaGLI1* exhibited positive transcriptional responses in the incompatible interaction, thereby providing reliable evidence that these genes participated in G3P synthesis and the defense response during wheat–*Pst* interaction. Although the cloned *TaGPDH1-3* encodes a G3P dehydrogenase, only *TaGPDH2* and *TaGPDH3* had less upregulation of the incompatible interaction than that of *TaGLY1*. Therefore, *TaGPDH1* is only involved in the fatty acid biosynthesis pathway. These results were consistent with the report of Chanda et al. [11], which revealed that two *G3Pdh* isoforms contributed to SAR in *Arabidopsis*. In addition, *GLI1* is required for NHR; higher levels of its transcripts were induced from 12 hpi to 24 hpi by non-host pathogen challenges [16,17]. Their studies suggested that the functional roles of *GLY1* and *GLI1* in SAR were due to their combined ability to reduce the G3P level. Although we identified four *TaG3Pdhs* and *TaGLI1*, wheat is a hexaploid plant that may possess more isoform genes.

*GLY1* and *GLI1* engage in two parallel pathways for G3P metabolism. Thus, the VIGS system was employed to individually or simultaneously analyze the role of *TaGLY1* and *TaGLI1* in G3P synthesis. In the current study, the necrotic areas triggered by infection of the avirulent pathogen were significantly different at 48 hpi in plants with knocked-down *TaGLY1*, *TaGLI1* and *TaGLY1*/*TaGLI1*, as compared with the control plants. According to previous microscopy studies on the incompatible interaction between Su11 wheat and *Pst*, HR is induced by the invasion of the avirulent *Pst* at 24 hpi, which becomes instantly extensive in the late stages of *Pst* infection [19]. These observations led us to propose that *TaGLY1* and *TaGLI1* are partly or indirectly associated with cell death. Furthermore, the areas of infection sites in leaves with gene silencing were significantly extended in the late stages of *Pst* invasion. Consistently, few *Pst* uredia were observed on wheat leaves infected with CYR23 at 14 dpi when *TaGLY1*, *TaGLI1* and *TaGLY1*/*TaGLI1* expression were knocked-down, thereby suggesting that the suppression of *TaGLY1* and *TaGLI1* transcripts could promote infection during hyphal growth and sporulation in plants. In addition, *TaGLY1* and *TaGLI1* were speculated to contribute to the inhibition of hyphal expansion and regulation of cell death by controlling the host G3P metabolism. As the substrate for GLI1, glycerol can be converted to DHAP for GLY1 catalyzed reaction *in vivo* [11]. In the present study, the results of histological observations and *Pst* uredia/leaf areas ratios also demonstrated that more obvious susceptible response in plants with knocked-down *TaGLY1* was observed than that in plants with knocked-down *TaGLI1*, indicating that GLY1 can partially revive the missing function of GLI1. Previously, Nandi et al. (2004) reported that *GLY1* is essential for SAR, but not for *P. syringae* basal resistance. However, Chanda et al. (2008) reported that a *gly1* mutant exhibited a compromised basal resistance to *C. higginsianum*. Similarly, we observed that VIGS of *TaGLY1* and *TaGLI1* compromised resistance to *Pst*. A possible explanation for this phenomenon is the large difference in the mechanisms of pathogenicity by the various pathogens.

Although previous studies imply that HR cell death is associated with SA-mediated defense signaling [20,21], this association remains unclear in wheat–*Pst* interaction. Local infection by a pathogen can further result in the immunization of the remaining foliage against subsequent infections, this phenomenon was phrased as SAR [13]. SAR requires the crucial signaling molecule SA, which is typically associated with the systemic expression of pathogenesis-related protein-coding genes and other putative defenses [22,23]. The VIGS results of the current study further supported that the reduction of *TaGLI1* and *TaGLY1* transcript levels contributed to the suppression of SA accumulation and *TaPR1* expression in response to the avirulent pathogen infection. The plants with co-silencing showed the superposition of these suppression abilities. The SA signaling pathway for activating disease resistance has been previously studied; SA accumulation is typically induced by *R* gene-mediated signaling (such as NDR1, EDS1, PAD4 and SAG101) [24]. Interestingly, *AtGLI1* is essential in the gene-for-gene resistance of NHR against *Pseudomonas* spp. [16]. Therefore, *TaGLI1* and *TaGLY1* may be involved in regulating the SA signaling pathway. These results are consistent with the report of Nandi et al. (2004), which revealed that the *gly1* mutation decreased the SAR-associated accumulation of elevated SA levels and the *PR1* gene transcript in *Arabidopsis* leaves. Their study implied that GLY1 is required for the transmission of mobile signal for SAR activation. By contrast, *gly1* and *gli1* plants did not have defective SA responsiveness after *P. syringae* inoculation, although the pathogen-induced G3P accumulation in *Arabidopsis* preceded the increased SA levels [11]. The most likely explanation is that G3P is associated with the transmission of SA, but it does not affect SA biosynthesis in plants [12].

To further analyze the functions of *TaGLY1* and *TaGLI1*, the BSMV-VIGS system was improved to verify the reliability of our results. We used two recombinant vectors from *TaGLY1* and *TaGLI1*, respectively, for co-silencing their target genes by VIGS. Meanwhile, percentage quantification of the uredia covering and histological statistical analyses showed subtle changes generated by the knockdown of target genes. In addition, qRT-PCR was applied to measure the efficiency of these changes. Therefore, the simultaneous co-silencing of *TaGLY1* and *TaGLI1* was found to be a feasible method to study their functions.

RNA interference (RNAi) has been a remarkable tool for knocking down the activity of specific genes, and gene silencing often depends on the transgenic technology to be integrated at different chromosomal locations [25–27]. Given that wheat has a relatively large genome, a high number of DNA repeat sequences, and a low regeneration ability, it is considered a recalcitrant plant for genetic transformation, as compared with other crops [28]. Several studies have shown that the unstable transformation system of wheat could be replaced by the VIGS technique, which has been used to perform both forward and reverse genetics to identify plant genes involved in several plant processes [29]. Previous reports showed that several genes can be simultaneously silenced by combinatorial RNAi in *Drosophila* and *Chlamydomonas* [30,31], thereby suggesting that co-silencing is a useful silencing strategy to characterize gene functions. Therefore, the co-silencing of two or more genes using the VIGS system proved to be a powerful reverse genetics tool for large-scale and high-throughput functional analyses of plant genes, particularly in plants which are difficult to genetically transform.

## Materials and Methods

### Plant materials and inoculation

Wheat cultivar Su11 and two *Pst* races, CYR23 and CYR31 were used in this study. Su11 displays a typical HR upon infection with CYR23, but is susceptible to CYR31. For biological stress treatments, the plants were grown, inoculated and maintained as previously described [32].

### RNA extraction, cDNA synthesis and quantitative real-time PCR

Total RNA was extracted using Trizol Reagent (Life Technologies, Grand Island, NY, USA) according to the manufacturer’s instruction. Genomic DNA contaminants were removed by DNase I treatment. First-strand cDNA was synthesized using the M-MLV reverse transcriptase (Promega, Shenzhen, China) with an oligo-(dT_18_) primer.

qRT-PCR was performed using a 7500 Real-Time PCR System (Life Technologies, Grand Island, NY, USA). The primers for qRT-PCR are listed in Table S1. Relative gene quantification was performed using the comparative 2^*–ΔΔC*^_*T*_ method [33] and normalized using the corresponding expression of the wheat elongation factor gene *TaEF-1a* (Genbank accession No. Q03033). All reactions were performed in triplicate, including three controls without the template.

### Sequence analysis, alignment and domain prediction of the deduced TaGLI1

Structural domains were annotated according to the Pfam (http://pfam.sanger.ac.uk/) and InterProScan (http://www.ebi.ac.uk/Tools/pfa/iprscan/) results. Multiple sequence alignment was accomplished using ClustalX (version 2.0). Phylogenetic analyses of TaGLY1 and TaGLI1 were performed using MEGA (version 5). The phylogenetic trees were constructed from the full-length amino acid sequences using the neighbor-joining method.

### BSMV-mediated TaGLI1 gene silencing

A 250-bp specific cDNA fragment of *TaGLY1* (Figure 4A) and a 258-bp specific cDNA fragment of *TaGLI1* (Figure 4B) with *Nhe*I restriction sites were obtained by reverse transcription PCR using oligonucleotide primers (Table S1) to construct the original BSMV:γ vector for gene silencing, as previously described [34]; the constructs were designated as BSMV:TaGLY1 and BSMV:TaGLI1, respectively. A BLAST search of the two fragments against the GenBank database did not identify wheat genes other than TaGLY1 and TaGLI1 sequences, thereby indicating the specificity of the sequence fragments. The capped *in vitro* transcripts were prepared from linearized plasmids containing the tripartite BSMV genome [35] using the RiboMAX^TM^ Large-Scale RNA Production System-T7 (Promega, Shenzhen, China) and the Ribo m^7^G Cap Analog (Promega, Shenzhen, China), according to the manufacturers’ instructions. The second leaves of the two-leaf-stage wheat seedlings were infected with BSMV by rub inoculation and lightly misted with DEPC-treated water. After incubation for 24 h in the dark, seedlings were placed in a growth chamber at (25 ± 2) °C. The fourth leaf of each plant was inoculated with urediniospores of CYR23 at 12 dpi. These leaves sampled at 0, 48 and 120 hpi for RNA isolation and qRT-PCR analysis. The infection phenotypes of stripe rust fungi were examined at 14 dpi. The experiment was repeated three times.

### Histological observations of fungal growth and host response

The *Pst*-inoculated leaves infected with BSMV were sampled at 48 and 120 hpi. The leaf samples were stained and fixed, as described by Wang et al. (2007). Infected mesophyll cells were observed to measure the necrotic death areas using epifluorescence microscopy based on the autofluorescence of the infected cells (excitation filter, 485 nm; dichromic mirror, 510 nm; and barrier filter, 520 nm). The lengths of the infective hyphae in *Pst* infection sites were measured under an Olympus BX-51 microscope (Olympus Corp., Tokyo, Japan). A minimum of five leaf segments were randomly selected for each treatment, and a minimum of 30 infection sites were examined.

### G3P and SA quantification

For G3P quantification, approximately 0.5 g fresh leaf tissue per sample was used to extract G3P, following the protocol described by Chanda et al. [11]. The extracts were analyzed with HPLC-MS (API 2000; AB SCIEX, Framingham, USA). For SA analysis, 100 mg to 200 mg fresh leaf tissue per sample was ground under liquid nitrogen and used to extract SA for HPLC-MS as previously described [36].

### Statistical analysis

Analysis of variance (ANOVA) was performed to determine the significant differences between each treatment using SAS (version 8.12; SAS Institute Inc., Cary, NC, USA). Fisher's LSD multiple range tests were used for multiple comparison tests.

### Accession Numbers

Genes mentioned in this article can be found in the GenBank data libraries under accession numbers as follows: At1g80460 (NM_106694), At2g40690 (NM_129631), At2g41540 (NM_129717), At3g07690 (NM_111648), At3g10370 (NM_111872), At5g40610 (NM_123425), Os01g58740 (AK073318), Os01g71280 (AK068569), Os01g74000 (AK070366), Os04g55410 (AK065350), Os05g41590 (AK101484), Os07g12640 (AK065591), *TaGLI1* (KC244204), *TaGLY1* (KC527592), *TaGPDH1* (KC953025), *TaGPDH2* (KC953026), *TaGPDH3* (KC953027), *TaPR1* (AAK60565) and *TaEF-1a* (Q03033).

## Supporting Information

Figure S1 **Multiple alignment and phylogenetic analysis of the predicted TaGLY1 amino acid sequence and other glycerol-3-phosphate dehydrogenases (G3PDH).** (A) Alignment of the predicted TaGLY1 amino acid sequence with G3PDH members in plants. Underline represents the NAD-binding domain. (B) A representative phylogenetic tree of TaGLY1 and G3PDH proteins in *Arabidopsis thaliana, Vitis vinifera, Populus trichocarpa, Ricinus communis, Glycine max, Sorghum bicolor, Zea mays, Oryza sativa* and *Brachypodium distachyon*. GeneBank accession numbers are provided after the gene names.
(PDF)

Figure S2 **Multiple alignment and phylogenetic analysis of the predicted TaGLI1 amino acid sequence and other glycerol kinases (GK).** (A) Alignment of the predicted TaGLI1 amino acid sequence with GK members in plants. Underline represents ATP-binding motif and asterisks represent glycerol binding sites. (B) A representative phylogenetic tree of TaGLI1 and GK proteins in *Glycine max, Medicago truncatula, Arabidopsis thaliana, Populus trichocarpa, Ricinus communis, Sorghum bicolor, Zea mays, Oryza sativa* and *Brachypodium distachyon*. GeneBank accession numbers are provided after the gene names.
(PDF)

Figure S3 **Relative transcriptional changes of other three wheat *GPDH* genes induced by *>Puccinia striiformis* f. sp. *tritici* infection in wheat leaves after inoculation with avirulent pathogen CYR23**. Leaf tissues were sampled for both inoculated and mock-inoculated plants at 0, 12, 24, 48, 72, and 120 hpi post inoculation. Relative expressions were calculated by the comparative threshold (2^-ΔΔ^ CT) method. The mean value and standard deviation expression were calculated from three independent biological replications.
(PDF)

Figure S4 **Quantification of the percentages of leaf areas covered with CYR23 uredia at 14 dpi when the transcriptions of *TaGLY1, TaGLI1,* and *TaGLY1/TaGLI1* were repressed.** The green&red images were converted from original images using the Adobe Photoshop software, and each pixel of images with same length leaf were categorized. Green represents wheat leaves and red represents *Pst* uredia.
(PDF)

Figure S5 **Relative transcript levels of *TaGPDH1-3* assayed in gene-knock-down wheat leaves in TaGLY1-silenced plants or TaGLI1-silenced plants, respectively or simultaneously in TaGLY1/TaGLI1-silenced plants at 0, 48 **and **120 hpi after inoculation with avirulent **pathogen CYR**23.** The mean value and standard deviation expression were calculated from three independent biological replications.
(PDF)

Table S1 **A list of PCR primers used in this work.**
(PDF)
