# Supplementary material for: Glycerol-3-Phosphate Metabolism in Wheat Contributes to Systemic Acquired Resistance against Puccinia striiformis f. sp. tritici
Source: PLoS One. 2013 Nov 29;8(11):e81756. doi: 10.1371/journal.pone.0081756 (PMC3843702; doi:10.1371/journal.pone.0081756)
Supplement: Figure S1 — Multiple alignment and phylogenetic analysis of the predicted TaGLY1 amino acid sequence and other glycerol-3-phosphate dehydrogenases (G3PDH). (A) Alignment of the predicted TaGLY1 amino acid sequence with G3PDH members in plants. Underline represents the NAD-binding domain. (B) A representative phylogenetic tree of TaGLY1 and G3PDH proteins in Arabidopsis thaliana, Vitis vinifera, Populus trichocarpa, Ricinus communis, Glycine max, Sorghum bicolor, Zea mays, Oryza sativa and Brachypodium distachyon. GeneBank accession numbers are provided after the gene names. (PDF) [file pone.0081756.s001.pdf]

Figure S1

A

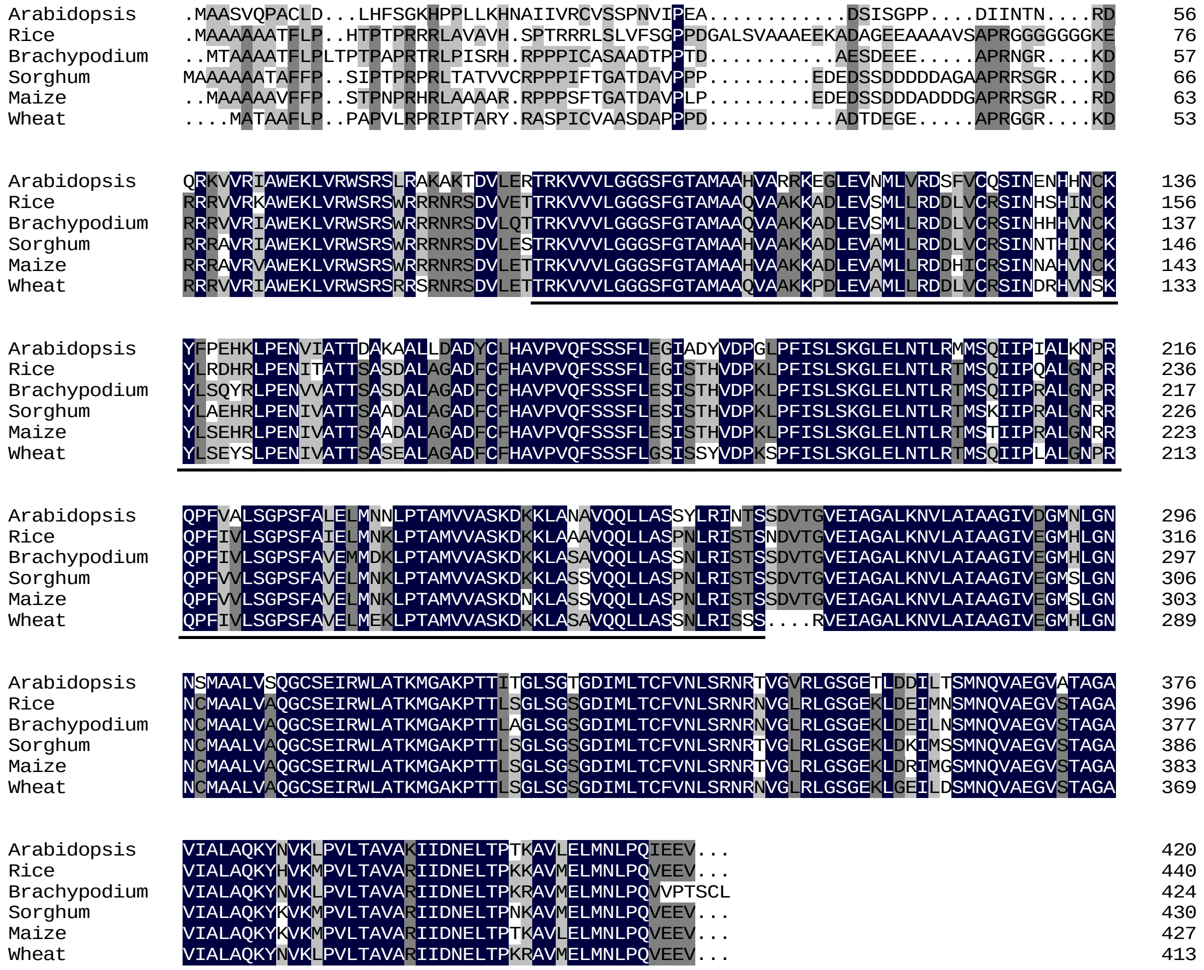

Arabidopsis .MAASVQPACLD...LHFSGKHPPLLKHNAIIVRCVSSPNVIPEA............DSISGPP....DIINTN....RD 56
Rice .MAAAAAATFLP..HTPTPRRRLAVAVH.SPTRRRLSLVFSGPPDGALSVAAAEEKADAGEEAAAAVSAPRGGGGGGGKE 76
Brachypodium ..MTAAAATFLPLTPTPAPRTRLPISRH.RPPPICASAADTPPTD...........AESDEEE.....APRNGR....KD 57
Sorghum MAAAAAATAFFP..SIPTPRPRLTATVVCRPPPIFTGATDAVPPP.........EDEDSSDDDDDAGAAPRRSGR...KD 66
Maize ..MAAAAAVFFP..STPNPRHRLAAAAR.RPPPSFTGATDAVPLP.........EDEDSSDDDADDDGAPRRSGR...RD 63
Wheat ....MATAAFLP..PAPVLRPRIPTARY.RASPICVAASDAPPPD...........ADTDEGE.....APRGGR....KD 53
Arabidopsis QRKVVRIAWEKLVRWSRSLRAKAKTDVLERTRKVVVLGGGSFGTAMAAHVARRKEGLEVNMLVRDSFVCQSINENHHNCK 136
Rice RRRVVRKAWEKLVRWSRSWRRRNRSDVVETTRKVVVLGGGSFGTAMAAQVAAKKADLEVSMLLRDDLVCRSINHSHINCK 156
Brachypodium RRRVVRIAWEKLVRWSRSWRRRNRSDVLQTTRKVVVLGGGSFGTAMAAQVAAKKADLEVSMLLRDDLVCRSINHHHVNCK 137
Sorghum RRRAVRIAWEKLVRWSRSWRRRNRSDVLESTRKVVVLGGGSFGTAMAAHVAAKKADLEVAMLLRDDLVCRSINNTHINCK 146
Maize RRRAVRVAWEKLVRWSRSWRRRNRSDVLETTRKVVVLGGGSFGTAMAAHVAAKKADLEVAMLLRDDHICRSINNAHVNCK 143
Wheat RRRVVRIAWEKLVRWSRSRRSRNRSDVLETTRKVVVLGGGSFGTAMAAQVAAKKPDLEVAMLLRDDLVCRSINDRHVNSK 133
Arabidopsis YFPEHKLPENVIATTDAKAALLDADYCLHAVPVQFSSSFLEGIADYVDPGLPFISLSKGLELNTLRMMSQIIPIALKNPR 216
Rice YLRDHRLPENITATTSASDALAGADFCFHAVPVQFSSSFLEGISTHVDPKLPFISLSKGLELNTLRTMSQIIPQALGNPR 236
Brachypodium YLSQYRLPENVVATTSASDALAGADFCFHAVPVQFSSSFLESISTHVDPKLPFISLSKGLELNTLRTMSQIIPRALGNPR 217
Sorghum YLAEHRLPENIVATTSAADALAGADFCFHAVPVQFSSSFLESISTHVDPKLPFISLSKGLELNTLRTMSKIIPRALGNRR 226
Maize YLSEHRLPENIVATTSAADALAGADFCFHAVPVQFSSSFLESISTHVDPKLPFISLSKGLELNTLRTMSTIIPRALGNRR 223
Wheat YLSEYSLPENIVATTSASEALAGADFCFHAVPVQFSSSFLGSISSYVDPKSPFISLSKGLELNTLRTMSQIIPLALGNPR 213
Arabidopsis QPFVALSGPSFALELMNNLPTAMVVASKDKKLANAVQQLLASSYLRINTSSDVTGVEIAGALKNVLAIAAGIVDGMNLGN 296
Rice QPFIVLSGPSFAIELMNKLPTAMVVASKDKKLAAAVQQLLASPNLRISTSNDVTGVEIAGALKNVLAIAAGIVEGMHLGN 316
Brachypodium QPFIVLSGPSFAVEMMDKLPTAMVVASKDKKLASAVQQLLASSNLRISTSSDVTGVEIAGALKNVLAIAAGIVEGMHLGN 297
Sorghum QPFVVLSGPSFAVELMNKLPTAMVVASKDKKLASSVQQLLASPNLRISTSSDVTGVEIAGALKNVLAIAAGIVEGMSLGN 306
Maize QPFVVLSGPSFAVELMNKLPTAMVVASKDNKLASSVQQLLASPNLRISTSSDVTGVEIAGALKNVLAIAAGIVEGMSLGN 303
Wheat QPFIVLSGPSFAVELMEKLPTAMVVASKDKKLASAVQQLLASSNLRISSS....RVEIAGALKNVLAIAAGIVEGMHLGN 289
Arabidopsis NSMAALVSQGCSEIRWLATKMGAKPTTITGLSGTGDIMLTCFVNLSRNRTVGVRLGSGETLDDILTSMNQVAEGVATAGA 376
Rice NCMAALVAQGCSEIRWLATKMGAKPTTLSGLSGSGDIMLTCFVNLSRNRNVGLRLGSGEKLDEIMNSMNQVAEGVSTAGA 396
Brachypodium NCMAALVAQGCSEIRWLATKMGAKPTTLAGLSGSGDIMLTCFVNLSRNRNVGLRLGSGEKLDEILNSMNQVAEGVSTAGA 377
Sorghum NCMAALVAQGCSEIRWLATKMGAKPTTLSGLSGSGDIMLTCFVNLSRNRTVGLRLGSGEKLDKIMSSMNQVAEGVSTAGA 386
Maize NCMAALVAQGCSEIRWLATKMGAKPTTLSGLSGSGDIMLTCFVNLSRNRTVGLRLGSGEKLDRIMGSMNQVAEGVSTAGA 383
Wheat NCMAALVAQGCSEIRWLATKMGAKPTTLSGLSGSGDIMLTCFVNLSRNRNVGLRLGSGEKLGEILDSMNQVAEGVSTAGA 369
Arabidopsis VIALAQKYNVKLPVLTAVAKIIDNELTPTKAVLELMNLPQIEEV... 420
Rice VIALAQKYHVKMPVLTAVARIIDNELTPKKAVMELMNLPQVEEV... 440
Brachypodium VIALAQKYNVKLPVLTAVARIIDNELTPKRAVMELMNLPQVVPTSCL 424
Sorghum VIALAQKYKVKMPVLTAVARIIDNELTPNKAVMELMNLPQVEEV... 430
Maize VIALAQKYKVKMPVLTAVARIIDNELTPTKAVLELMNLPQVEEV... 427
Wheat VIALAQKYNVKLPVLTAVARIIDNELTPKRAVMELMNLPQVEEV... 413

B

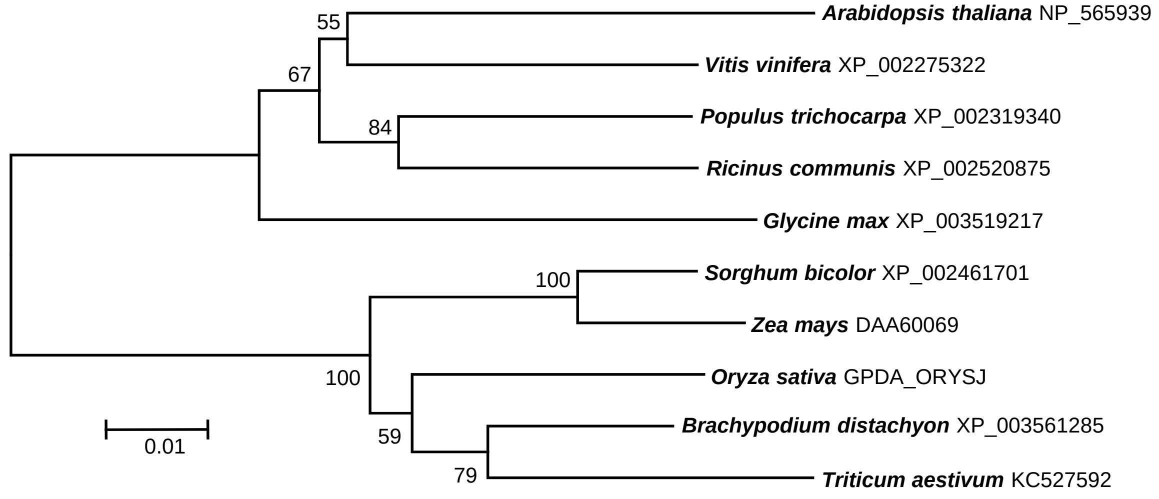

55
67
84
100
100
59
79
0.01
Arabidopsis thaliana NP_565939
Vitis vinifera XP_002275322
Populus trichocarpa XP_002319340
Ricinus communis XP_002520875
Glycine max XP_003519217
Sorghum bicolor XP_002461701
Zea mays DAA60069
Oryza sativa GPDA_ORYSJ
Brachypodium distachyon XP_003561285
Triticum aestivum KC527592
