# Supplementary figures and images for: Glycerol-3-Phosphate Metabolism in Wheat Contributes to Systemic Acquired Resistance against Puccinia striiformis f. sp. tritici
Source: PLoS One. 2013 Nov 29;8(11):e81756. doi: 10.1371/journal.pone.0081756 (PMC3843702; doi:10.1371/journal.pone.0081756)

Figure S2

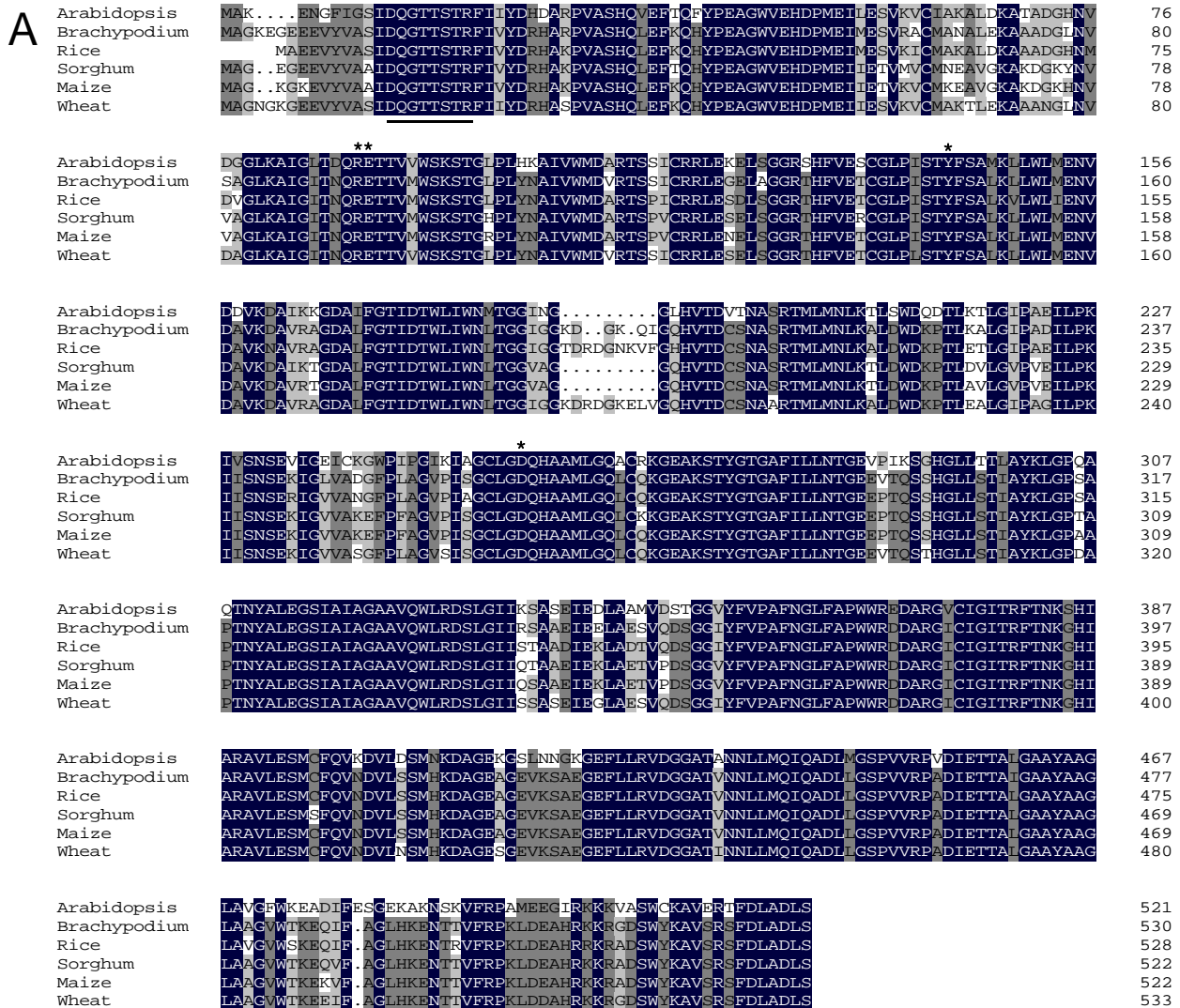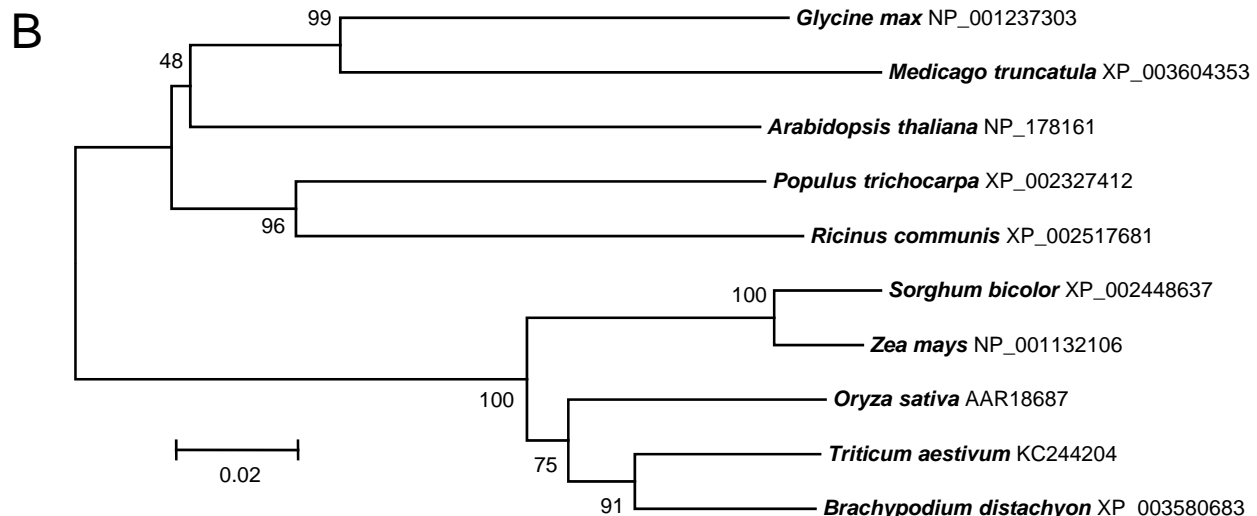

Supplement: Figure S2 — Multiple alignment and phylogenetic analysis of the predicted TaGLI1 amino acid sequence and other glycerol kinases (GK). (A) Alignment of the predicted TaGLI1 amino acid sequence with GK members in plants. Underline represents ATP-binding motif and asterisks represent glycerol binding sites. (B) A representative phylogenetic tree of TaGLI1 and GK proteins in Glycine max, Medicago truncatula, Arabidopsis thaliana, Populus trichocarpa, Ricinus communis, Sorghum bicolor, Zea mays, Oryza sativa and Brachypodium distachyon. GeneBank accession numbers are provided after the gene names. (PDF) [file pone.0081756.s002.pdf]

Figure S3

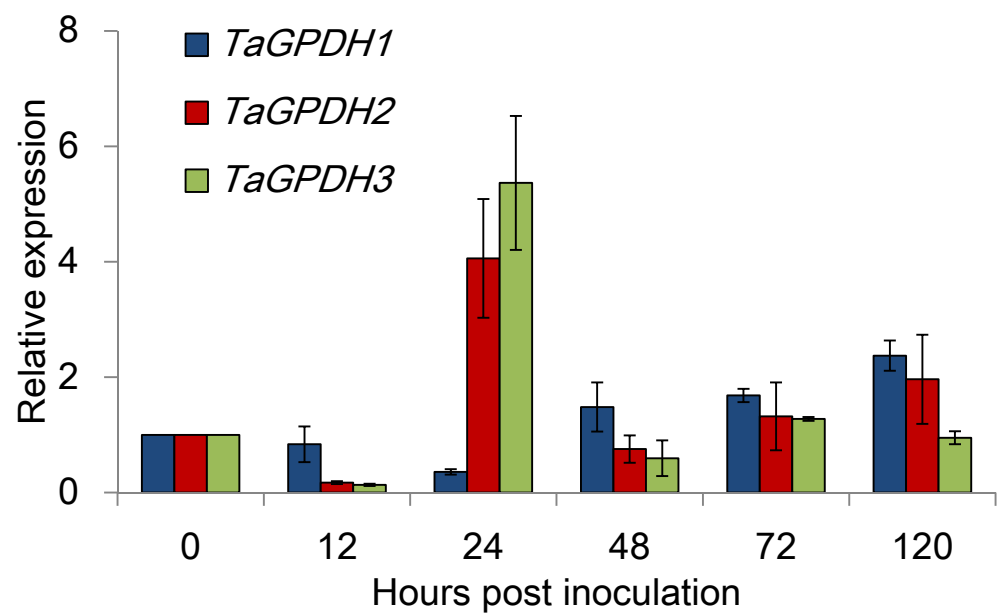

Supplement: Figure S3 — Relative transcriptional changes of other three wheat GPDH genes induced by >Puccinia striiformis f. sp. tritici infection in wheat leaves after inoculation with avirulent pathogen CYR23. Leaf tissues were sampled for both inoculated and mock-inoculated plants at 0, 12, 24, 48, 72, and 120 hpi post inoculation. Relative expressions were calculated by the comparative threshold (2-ΔΔ CT) method. The mean value and standard deviation expression were calculated from three independent biological replications. (PDF) [file pone.0081756.s003.pdf]

Figure S4

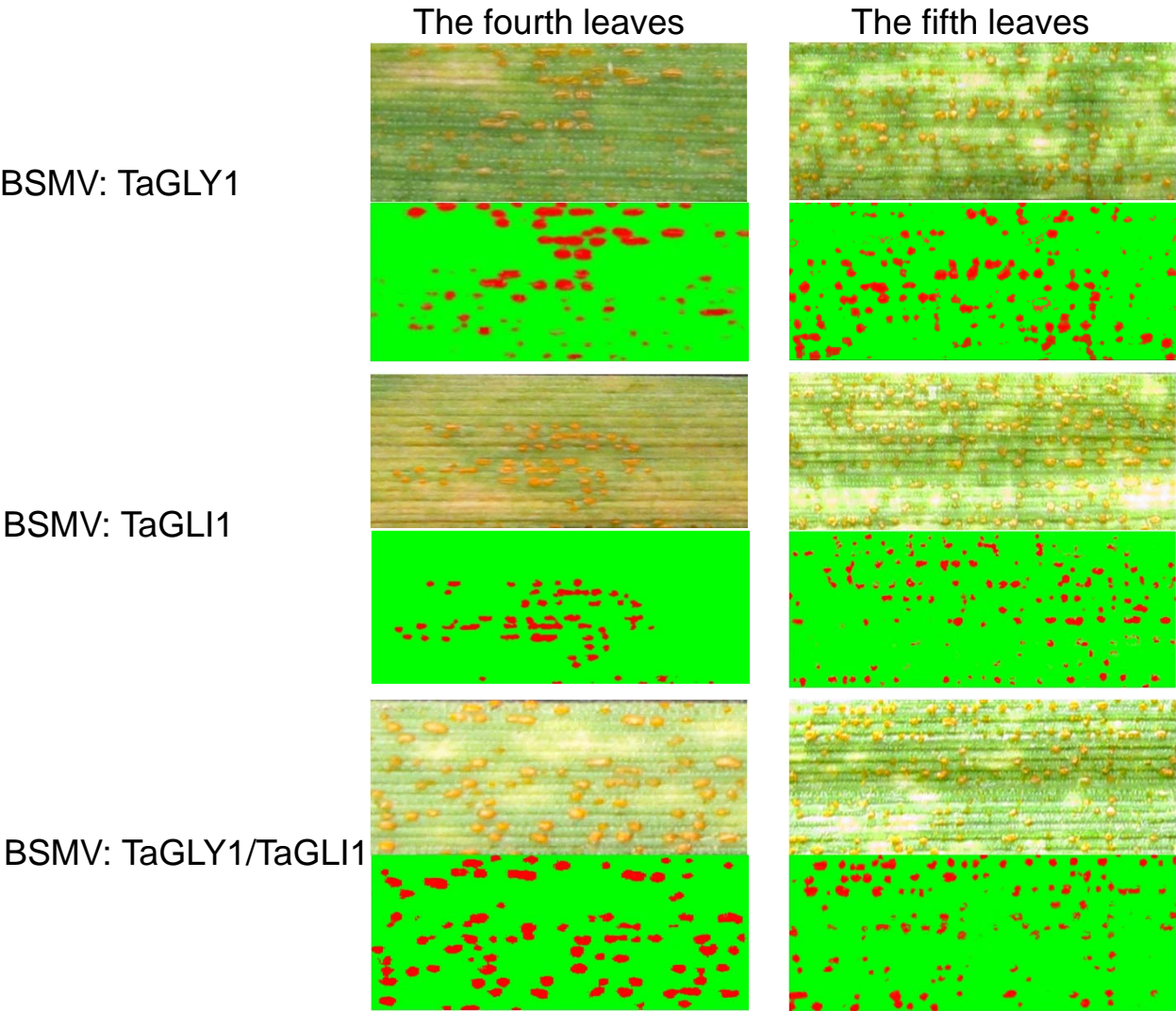

Supplement: Figure S4 — Quantification of the percentages of leaf areas covered with CYR23 uredia at 14 dpi when the transcriptions of TaGLY1, TaGLI1, and TaGLY1/TaGLI1 were repressed. The green&red images were converted from original images using the Adobe Photoshop software, and each pixel of images with same length leaf were categorized. Green represents wheat leaves and red represents Pst uredia. (PDF) [file pone.0081756.s004.pdf]

Figure S5

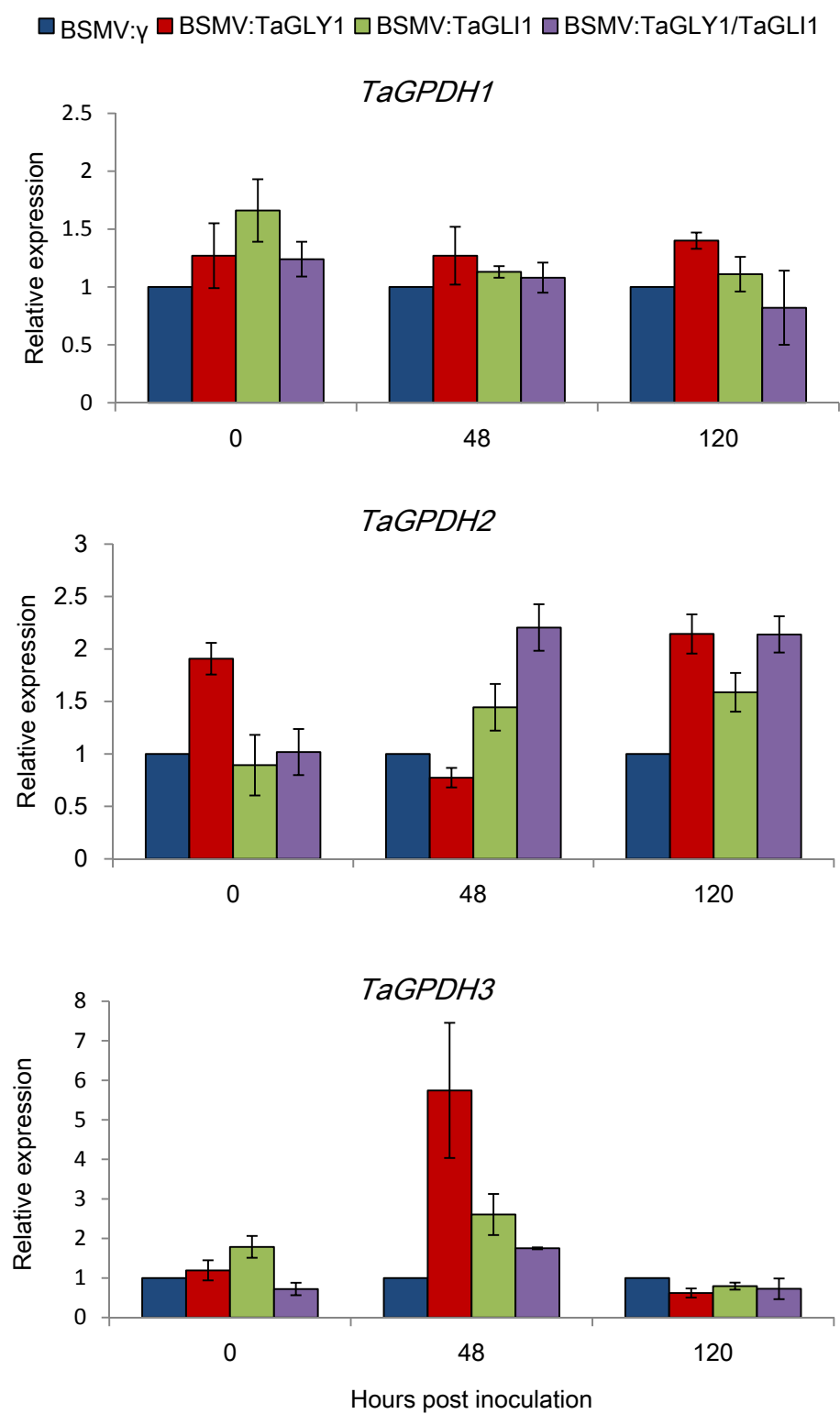

Supplement: Figure S5 — Relative transcript levels of TaGPDH1-3 assayed in gene-knock-down wheat leaves in TaGLY1-silenced plants or TaGLI1-silenced plants, respectively or simultaneously in TaGLY1/TaGLI1-silenced plants at 0, 48 and 120 hpi after inoculation with avirulent pathogen CYR23. The mean value and standard deviation expression were calculated from three independent biological replications. (PDF) [file pone.0081756.s005.pdf]
