# Supplementary material for: Glycerol-3-Phosphate Metabolism in Wheat Contributes to Systemic Acquired Resistance against Puccinia striiformis f. sp. tritici
Source: PLoS One. 2013 Nov 29;8(11):e81756. doi: 10.1371/journal.pone.0081756 (PMC3843702; doi:10.1371/journal.pone.0081756)
Supplement: Table S1 — A list of PCR primers used in this work. (PDF) [file pone.0081756.s006.pdf]

Table S1 A list of PCR primers used in this work.

Primer name	Primer sequence (5’-3’)	Usage
TaGPDH1-F	ggggactggagtgtttagg	Amplification of *TaGPDH1* sequence by RT-PCR
TaGPDH1-R	tgacagggttggagctaat	
TaGPDH2-F	acgactctcgtttgaatg	Amplification of *TaGPDH2* sequence by RT-PCR
TaGPDH2-R	tgtactggagcctgcttt	
TaGPDH3-F	cggtctgtcctccctgtt	Amplification of *TaGPDH3* sequence by RT-PCR
TaGPDH3-R	agcccaaattgttcttgatg	
TaGLY1-F	aaccgagaaaggggcggaacg	Amplification of *TaGLY1* sequence by RT-PCR
TaGLY1-R	accaagacgcaatcccaca	
TaGLI1-F	cttgttccaccggctcactc	Amplification of *TaGLI1* sequence by RT-PCR
TaGLI1-R	ctttctgaagaatccagatgttcct	
TaGPDH1-QF	attggtgctggaatggtggc	Quantitative RT-PCR of *TaGPDH1* transcripts
TaGPDH1-QR	ggctgttctgtcagtagatgggtaa	
TaGPDH2-QF	cataacgggcaatgactgaaaa	Quantitative RT-PCR of *TaGPDH2* transcripts
TaGPDH2-QR	gtgtagacagggaaaacaaaaggat	
TaGPDH3-QF	cttctgttcccttcagtcagag	Quantitative RT-PCR of *TaGPDH3* transcripts
TaGPDH3-QR	gcttctgccactctcctattt	
TaGLY1-QF	ggagcaaagccaacaaccc	Quantitative RT-PCR of *TaGLY1* transcripts
TaGLY1-QR	accaagacgcaatcccaca	
TaGLI1-QF	tcaagcagcactacccg	Quantitative RT-PCR of *TaGLI1* transcripts
TaGLI1-QR	caaaccagcatccacatta	
TaPR1-QF	gagaatgcagacgcccaagc	Quantitative RT-PCR of *TaPR1* transcripts
TaPR1-QR	ctggagcttgcagtcgttgatc	
TaEF-1a-QF	tggtgtcatcaagcctggtatggt	The reference gene for quantitative RT-PCR
TaEF-1a-QR	actcatggtgcatctcaacggact	
TaGLY1-VF	ctaGCTAGCttgcgtcttggttcaggc	Construction of BSMV:TaGLY1 for silencing *TaGLY1*
TaGLY1-VR	ctaGCTAGCcggtggctccattctttt	
TaGLI1-VF	ctaGCTAGCgagtcaggagaagtgaagagtgc	Construction of BSMV:TaGLI1 for silencing *TaGLI1*
TaGLI1-VR	ctaGCTAGCtgcgtcatctaacttgggg	

The underlined nucleotides indicate *Nhe*I (GCTAGC) restriction site.
